# Uncovering the moral heuristics of altruism: A philosophical scale

**DOI:** 10.1371/journal.pone.0229124

**Published:** 2020-03-23

**Authors:** Julian Friedland, Kyle Emich, Benjamin M. Cole

**Affiliations:** 1 College of Business, Metropolitan State University of Denver, Denver, Colorado, United States of America; 2 Lerner College of Business & Economics, The University of Delaware, Newark, Delaware, United States of America; 3 Gabelli School of Business, Fordham University, New York, New York, United States of America; University of California Los Angeles, UNITED STATES

## Abstract

Extant research suggests that individuals employ traditional moral heuristics to support their observed altruistic behavior; yet findings have largely been limited to inductive extrapolation and rely on relatively few traditional frames in so doing, namely, deontology in organizational behavior and virtue theory in law and economics. Given that these and competing moral frames such as utilitarianism can manifest as identical behavior, we develop a moral framing instrument—the Philosophical Moral-Framing Measure (PMFM)—to expand and distinguish traditional frames associated and disassociated with observed altruistic behavior. The validation of our instrument based on 1015 subjects in 3 separate real stakes scenarios indicates that heuristic forms of deontology, virtue-theory, and utilitarianism are strongly related to such behavior, and that egoism is an inhibitor. It also suggests that deontic and virtue-theoretical frames may be commonly perceived as intertwined and opens the door for new research on self-abnegation, namely, a perceived moral obligation toward suffering and self-denial. These findings hold the potential to inform ongoing conversations regarding organizational citizenship and moral crowding out, namely, how financial incentives can undermine altruistic behavior.

## Introduction

Mounting evidence suggesting that moral heuristics may underlie observed altruistic behavior has fallen broadly within an organizational behavior approach rooted in deontic justice theory [1–2] and a law and economics approach rooted in virtue theory [3–5]. Both research streams support the view that individuals employ non-egoistic moral framing to back their altruistic behavior, thus moving beyond the *homo economicus* paradigm. However, up to now, these literatures have largely been limited to extrapolating philosophical frames from observed behavior. Thus, for example, when organizational scholars observe adults behaving seemingly altruistically in economic dictator games, it has been characterized as motivated by internalized deontic notions of justice as fairness (e.g., [1–2,6]). Recent scholarship has suggested that such extrapolations may be too narrow as non-self-interested behavior need not be deontic, given that other traditional frames such as utilitarianism and virtue theory could yield identical behavioral outcomes [7]. And some research has found a distinction between inequality aversion and joint gain maximization [8–9] which could indicate deontic versus utilitarian outlooks. Furthermore, many have extrapolated that self-interested behavior may be conditioned by egoistic philosophical framing [5,7,10]. Our findings provide quantitative support for these qualitative suspicions.

If, as the evidence suggests, moral heuristics interact with other factors in promoting or inhibiting altruistic behavior, there is a need for developing effective tools for measuring the moral framing underlying and impeding altruistic behavior, in order to (1) avoid incorrect extrapolations of observed behavior and (2) uncover how best to crowd it into organizational settings without necessarily resorting to economic incentives or empathic priming alone. Our instrument offers a way forward in these directions by providing a frame-specific measure of traditional moral motivational frames as they appear heuristically in practice.

In this paper, we detail the development of a Philosophical Moral-Framing Measure (PMFM), to distinguish between competing moral frames that may underlie actor behavior. Philosophers—and increasingly psychologists—traditionally divide the moral landscape into four overarching theoretical frames, namely, deontology [11], utilitarianism [12], virtue theory [13], and egoism [14], though it should be noted that egoism is most often treated in the philosophical literature as a description of human behavior than as a prescriptive moral theory, *per se*. Still, given its longstanding academic and educational influence in fields such as business and economics, it stands in as a prescriptive theoretical point of view. As such, it is treated here more as a counterfoil than as a full-blown moral theory. It should be noted that this is not intended as an exhaustive list of moral frames, but only representative of the three overarching ethical frameworks (plus egoism) most widely taught in standard secondary and post-secondary contexts. Furthermore, we have chosen to limit our study to exclude political theory, such as social contract theory and libertarianism though there will certainly be overlap into this normative arena. See for example [15] for a novel articulation of contractualism as an ethical theory in its own right.

In the sections that follow, we provide background regarding the intellectual and philosophical underpinnings of these “Big 4” theoretical frames, describing the normative connection between each frame and altruistic behavior. We then detail the scale items through which we set about to measure people’s general adherence to these respective frames in three empirical studies using four unique samples. Items loaded mostly as expected while revealing systematic overlap between deontology and virtue theory and suggesting the possibility of a fifth moral frame previously not fully studied. Data gathered during scale validation make five significant contributions to our understanding of general moral framing and to our understanding of its connection to altruistic behavior: (1) indicating that adults hold heuristic versions of traditional philosophical frames known as deontology, virtue theory and utilitarianism, and (2) that these are all predictive of altruistic behavior, (3) indicating that egoism may inhibit such behavior, (4) providing new evidence that deontology and virtue-theory may be generally perceived as intertwined, and (5) opening the door for new research on self-abnegation, or a moral obligation toward suffering and self-denial.

## Theoretical development

### *Homo economicus* and moral crowding out

The introduction of *homo economicus* as a way of explaining human behavior has spurred decades of research to counter what has been characterized as an undersocialized [16–17], unrealistic [18] and incomplete [19] representation of a complex animal—the human actor. On one side, *homo economicus* left no room for cognitive limitations [20–21]; on the other, it left no room for contemplation [22], for electing actions for reasons beyond that of reductionist personal gain. In fact, the traction *homo economicus* experienced in the 20th century as an instrument for understanding actor behavior is curious, given that philosophers have struggled with understanding human action for centuries before *homo economicus* entered academic parlance. Plato, Aristotle, Kant, Smith, Mill, and Nietzsche are household names precisely because they articulated characterizations and rationales that have stood the test of time. Despite the longevity of these thinkers’ ideas, organizational scholars seem to have gravitated toward considering only a small number of extrapolated normative rationales of observed outcomes.

Furthermore, educational exposure to neoclassical economic theory reifying the narrow *homo-economic* conception of human nature, characterizing human behavior as motivated by instrumental and relational self-interest such as financial gain, social status, and political advantage [14,23], has been shown to substantially increase self-interested behavior [10,24–26]. Ultimately, the ubiquity of this framing within organizations has created a cultural environment in which financial incentives may crowd out altruistic behavior [4–5]. Indeed, the continuing preponderance of high-profile ethics scandals from Volkswagen and Wells Fargo to Amazon and Facebook is causing concern that the global push toward freer markets fueled by such drives may not maximize human flourishing and social welfare externalities [27–28].

There is some indication that altruism can be restored to an extent via empathic priming [29–30] or experiences of awe inducing a small sense of self [22]. Unfortunately, once altruistic dispositions fade, they are difficult to re-instill over the long term through empathic priming alone. Empathy may compromise moral decision-making [31] and individuals may end up experiencing compassion fatigue [32]. This raises the question of the extent to which moral framing together with empathic priming might help *crowd-in* altruistic behavior more durably than empathic priming alone. Philosophical appeals to civic pride have been shown to be effective in inspiring altruistic behavior (e.g., [33–36]). Self-image has been shown to be morally motivating [37], playing a key role in stimulating altruistic behavioral development [28] and subjects will only cheat to the extent that they can still maintain a belief in themselves as non-cheaters [38]. So while empathy and awe largely function as pre-theoretical behavioral drivers, even they may be buttressed or inhibited by moral framing. In other words, agents may hold philosophical reasons for behaving more or less self-interestedly or as a result of empathy and/or awe depending on acknowledged or internalized theoretical frames. Similarly, persistent moral and political differences have been traced to opposing conceptual frames [39–40]. The current paper is motivated by our belief that scholars must toss a wider net to understand the ethical motivations of seemingly altruistic behavior, and need better tools to do so rigorously. Additionally, it is important to compare and contrast the role of traditional philosophical frames in driving—and developing—moral behavior. While philosophers generally have discussed four major theoretical frames of moral motivation, it is still unclear the extent to which laypersons recognize and use heuristic versions of these frames when making every day moral decisions such as whether or not to behave altruistically, though there is new evidence that virtue theory, deontology, and consequentialist heuristics influence moral judgments in hypothetical high-stake scenarios [41]. In the sections that follow, we focus on the Big 4 theoretical frames of moral motivation—deontology [11], utilitarianism [12,42], virtue theory [13], and egoism [14,43]. We then detail specific items used to distinguish these four frames as unique. Finally, we discuss the expected and unexpected results of our exploratory and confirmatory factor analyses, and how our instrument might help scholars to reliably distinguish these important moral frames and practitioners to harness their potential to foster positive civic and organizational behavior.

### Theoretical frames

#### Deontology

Deontology is etymologically defined as the logic of duty. This means that what is good is taken to be a matter of strict rule-based principle and not of consequences. In other words, the ends do not justify the means. Moral rules are categorical and do not allow for exceptions. Thus, by implication, the frame concerns itself less with performing good deeds than with avoiding immoral ones. The theory, originally attributed to German philosopher Immanuel Kant and recently rearticulated by Derek Parfit [44], takes the Good to be an objective aspect of reality governed by the logic of universalizability, meaning all good acts must be logically universalizable and not self-defeating were everyone to act in a given manner [45]. For example, deception is seen as always wrong since if everyone were to lie when it benefitted him or her, there would be no more advantage to be derived from deception because trust—the social glue that liars exploit—would evaporate. Thus, any action is immoral and unjust if it makes itself logically more difficult and ultimately impossible to carry out the more people indulge in it. Such behavior is not only self-defeating but also threatens the sustainability of the social system by putting the entire bedrock of public reason in jeopardy [44]. Fairness is taken to be an implication of this frame, since rules must apply to everyone equally for the logic of universalizability to function [46].

Despite the fact that it is not traditionally characterized as goal-directed, the deontic moral frame nevertheless requires selfless behavior as agents must forego potential personal gains from engaging in so-called white lies–or indeed any exception to any moral rule whatsoever that could bring about positive results to those concerned. It is important to note that while this theory most closely embodies the “Golden Rule” maxim of doing onto others as you would have them do onto you, it is not rooted primarily in empathy for others. Nevertheless, a measure of empathic sensibility is essential to behaving ethically [47], and Kant’s “kingdom of ends” formulation has been shown to allow conscientious deontologists to foresee whether their actions would inflict harm [48]. As such, instead of considering the goodness of actual results, deontology takes agents’ intentions as essential to determining the goodness of their actions. Ultimately, ethical imperatives are dictated by our own self-consciousness and rational rule-based normativity aimed at emulating the best possible world. It is our rational ability to acknowledge our own conscience together with the logical fabric of the Good that makes each of us into freely autonomous persons or “ends in ourselves” as Kant puts it. As such, it is irrational and thence immoral to neglect or exploit rational beings, for to do so would be to treat them as pawn-like objects with no intrinsic aims of their own. Kant, therefore, argues that any rational being should expect to receive reasonable altruistic consideration in times of basic need, so long as others are materially disposed to provide it without thereby sacrificing their own basic needs [45].

#### Utilitarianism

Utilitarianism defines the Good as what is most “useful” for achieving the greatest happiness for the greatest number, where happiness is taken to be a form of pleasure and absence of pain [49]. As such, and counter to deontology, it is often taken to be synonymous with consequentialism because the end results of our actions are all that ultimately matter. It is, therefore, entirely goal-directed, or “teleological” in philosophical terms. Utilitarianism as a theory is most closely associated with the British enlightenment, originating in Scotland with David Hume [50] and Adam Smith [42], and further developed by English philosophers Jeremy Bentham [49], John Stuart Mill [12], and Henry Sidgwick [51]. It is arguably the most influential theory in applied ethics in the English language and is espoused by the most prominent applied ethicist alive today, Peter Singer [52]. Utilitarians believe everyone’s interests should be considered equally and that achieving the greatest balance of pleasure over pain for all is the absolute aim. While there exist more sophisticated forms of utilitarianism, including rule-utility and preference-utility, we are confining our account to the classical act-utilitarian theory as expounded by Jeremy Bentham.

Utilitarianism is particularly helpful in situational dilemmas in which costs and benefits must be tabulated in order to arrive at the most “optimific” outcome for all concerned [53]. Although its adherents take it to be just as egalitarian as deontology, it is not as deeply rooted in fairness because it holds fast to the notion that the ends always justify the means. Thus, for example, it may be necessary at times to inflict lesser harms to some so as to avoid far greater harms to others—a line of thinking the deontic frame categorically rules out. Utility, by definition, admits no categorical rules since any action is theoretically permitted in some possible scenario [12].

At the same time, the utilitarian frame is extraordinarily selfless since it requires that we always choose whatever action works to the greatest happiness of the greatest number. No one can ever privilege one’s own position over anyone else’s unless doing so would increase total happiness. For in the words of Jeremy Bentham, “each is to count for one and none for more than one” [49: Corollary 1, Ch.17]. Furthermore, since happiness is the ultimate aim, the theory is generally interpreted as relying more on emotion than deontology. Indeed, the first premise upon which the theory rests, namely, that we should seek to realize the greatest good for the greatest number, functions as an emotive appeal to shared happiness. Still, reason is of course required to carry out the hedonistic calculations for determining the best course of action in any given context. And since all interests are to be considered equally, a dispassionate attitude is required in order to conduct unbiased calculations of which actions are ultimately preferable to all concerned.

Recent work suggests that a two-dimensional model of utilitarian psychology may exist—in lay respondents but not moral philosophers—opposing impartial beneficence and instrumental harm when considering sacrificial dilemmas [54]. Although we do not explore this distinction here since our scale is not designed to account for how agents attempt to resolve extraordinarily challenging sacrificial dilemmas in which there is no entirely satisfactory solution, we consider possible overlap in our Surprise Findings on self-abnegation discussed below.

#### Virtue theory

Virtue theory conceptualizes the Good as a natural developmental function of all living things. As such, it is defined psychologically as that at which all things aim, namely, self-actualization [55]. It is the oldest of the moral frames, originating in Ancient Greece, most notably in the works of Plato and especially Aristotle. Its approach is uniquely psychological, as it is chiefly concerned with the question of what makes a person good as opposed to what makes an action good; the latter are questions deontology and utilitarianism confront more directly. Since it is the oldest of the moral frames, much of it is embedded within the other more recent frames through historical influence. For instance, self-actualization is generally defined as happiness, though unlike utilitarianism, which takes this as synonymous with pleasure, virtue theory defines happiness more specifically as an ongoing aspirational process of personal development best referred to as human flourishing [55].

For virtue theory, for example, learning to excel at piano would be a kind of happiness, but not merely a pleasurable feeling as with utilitarianism. Rather, happiness in this sense is construed as an activity and overarching function of a good life. Nevertheless, many utilitarians (following Mill) take virtue theory to be a kind of utilitarianism because of its emphasis on maximizing happiness [56]. And while the two approaches do have this teleological aspect in common, they are not generally considered identical nor necessarily compatible. Unlike utilitarianism, virtue theory is focused first and foremost on the psychology of the good person and does not reduce the Good to the experience of pleasure. As such, what makes an action ‘good’ is measured less by how it affects the world, than how it shapes or reveals the character of the individual engaging in it. It is therefore not generally used as a synonym for consequentialism since it does not focus mainly on the material consequences or one’s actions.

Still, virtue theory, like deontology and utilitarianism, is highly other-regarding. This is because it sees virtuous development to be impossible in isolation and takes eusociality as a fundamental condition of human nature. Humans are considered to be social beings and the good life is thereby only achievable in harmonious relation with others [55]. For this reason, persons who only consider their own interests are taken to be conflicted and self-loathing, and can never be considered truly fulfilled [55]. This is because the frame takes the Good as a natural function of psychological health. Hence, it follows logically from this premise that immorality can only occur if the agent is somehow deceived or delusional. Otherwise, any sane person would be compelled to do the right thing in every situation. Thus, if immoral persons claim to be happy, they are necessarily blinded or confused about the actual nature of both the Good and happiness, which are two sides of the same coin. Intriguingly, a growing sociobiological literature suggests that altruism has been biochemically embedded as a group-level adaptive trait in myriad species defined as eusocial, including our own [57].

The final notable aspect of virtue theory is its conception of temperance (moderation) as the most valuable psychological disposition [55]. This stems from the fact that immoral acts are seen as the result of placing excessive or deficient importance on certain desires, which is the ultimate result of moral ignorance and its attendant unhappiness.

#### Egoism

Egoism is defined classically as the self-interested point of view. Actors motivated by this frame only act to benefit themselves materially and socially, which means that other-regarding behavior is only undertaken when it instrumentally advantages the actor in some way. Thus, while the frame is teleological in the goal-directed or consequentialist sense, genuine altruism is by definition impossible within it, making it fundamentally incompatible with utilitarianism. While psychological egoism has been influential in evolutionary theory [58] and economics [59], ethical egoism has had few adherents in the philosophical literature. While some may postulate that Adam Smith, inventor of the ‘invisible hand’ theory, was an adherent of ethical egoism, Smith was, in fact, an avowed proponent of Aristotelian virtue theory [60], only defending the profit motive as a more effective economic driver than benevolence [42]. As such, Smith is a hybrid thinker who tends toward utilitarianism in economic matters and virtue theory in non-economic matters. The German philosopher Friedrich Nietzsche is perhaps the only great thinker most directly associated with egoism in both ethical and psychological forms [43]. According to his account, human behavior is primarily motivated by the will to power [61]. Still, Nietzsche was also heavily influenced by Aristotelian virtue theory, recognizing human nature as social [62]. Yet neither Nietzsche nor Aristotle can be considered egalitarians; each believed some persons to be intrinsically superior and thus more meritorious than others.

Ethical egoism is hence never a direct motivator of altruistic behavior. It is not necessarily antisocial, however, since apparent altruistic behavior will still be carried out either cynically or unwittingly as an indirect means of leveraging an advantage over others. Still, when taken to its extreme, psychological egoism can become sociopathy, namely, the clinical inability to experience empathy—and there is some suspicion that an egoistic corporate culture has more than doubled the proportion of executives with sociopathic tendencies in corporate America than in the wider population [63–64].

### Key differences from existing instruments

There already exist a number of well-validated moral reflection scales measuring philosophical heuristics, the earliest being the 12-item Socio-Moral Reflection Measure (SRM) devised by Kohlberg and employed chiefly as an instrument for measuring the moral development of children [65], adolescents [66] and those with developmental or intellectual disabilities [67]. There is also the nearly identical but less time-consuming Defining Issues Test (DIT) which replaces the written scenario analysis format of the SRM with an item-recognition questionnaire on a Likert scale [68]. While the Kohlbergian developmental framework has obvious points of contact with the PMFM, there are significant differences between them. The main disadvantage of the SRM and DIT is that they do not provide adequate means to distinguish differing moral frames and may contain a deontic theoretical bias [7,69]. They take the deontic frame as the sixth and highest stage of development while placing consequentialism—a synonym for utilitarianism—lower down at stage five [65]. Moreover, they confine consequentialism to a contractualist linguistic frame (i.e., social convention) instead of the broader utilitarian theoretical aim of the most optimal outcome for all concerned [12]. This may be a legacy of Kohlberg’s preference for deontic morality [70] or a residual of cultures still steeped in Judeo-Christian norms. This deontically-inspired framework conceives the agent as a disengaged observer and as a result does not attempt to measure a virtue-ethical mindset in which doing the right thing is taken to be an act of self-fulfillment [71]. The construct also assumes egoism to be at the second lowest stage of development, whereas adults may in fact have sophisticated rationales for espousing such values.

It should be noted that Rest, the creator of the DIT, hypothesized contra Kohlberg that moral reasoning processes are distinct from motivation and implementation processes [68] and there has been some data to support the contention [72]. Still, we maintain there is good reason to expect that moral reasoning guides motivation to a significant extent, as the recent work of Rand and colleagues for example, reveals a significant role for deliberation in altruism and the extent to which subjects rely on it as opposed to intuition as conditioned by gender role attributes [73]. The data that you and Bebeau [72] supply on motivation is based on hypothetical professional scenarios that raise complex issues of power and conflicting moral and personal values. It is not surprising that in such cases, subjects may hesitate to expose themselves to do what they believe would be the right thing. In other contexts, such as those of our study where agents are less vulnerable, they will be more capable of acting in accordance with their ideals. Furthermore, philosophical appeals to virtue have been found throughout history to be morally motivating, indeed at times more so than financial incentives [4,74–75] or legal sanctions [36] in civic contexts. Philosophical values in leadership have been shown to influence employee beliefs and behaviors [76]. And the utilitarian philosophy of effective altruism is currently inspiring countless individuals to act in ways that maximize their positive impacts on the wider world [77]. Indeed, our findings suggest that moral framing along such lines may underlie seemingly-altruistic behavior.

There also exists the 8-point Multidimensional Ethics Scale (MES), [78] and a revised 14-point version (RMES) that seeks to measure deontology, utilitarianism, and egoism [79] under hypothetical scenarios of potential injustice. Although these scales suggest adults may think philosophically about justice, neither was adequate for our purposes for two reasons. Firstly, the PMFM is written specifically to measure broad philosophical norms undergirding actual altruistic behavior as opposed to evaluating hypothetical cases of potential injustice, which is the central aim of the MES and RMES. Secondly, it does not include a key aspect distinguishing utilitarianism and deontology, namely, the commitment to behaving logically. Indeed, this is the essential justificatory component of deontology [11,44] that distinguishes it from the utilitarian frame, which is justified entirely by emotion [49]. Thirdly, our tool also measures virtue-theoretical framing, while the MES and RMES do not. There are however some similarities between the PMFM and the RMES regarding deontology, utilitarianism, and egoism even though they cannot always be used interchangeably. Altruism has also been shown to be associated with ‘bright-sided’ personality traits on the Motives, Values, Preferences Inventory (MVPI) scale, which does not measure philosophical framing [80].

Finally, there exists a 6-factor virtue ethics scale [81], which measures 6 distinct virtues. However, virtues considered solely in themselves do not necessarily amount to a unique construct absent an overarching frame through which virtue itself is conceptualized. What essentially characterizes the virtue-theoretical outlook in the philosophical literature and distinguishes it from other frames is its conception of the Good as an aspirational activity of self-actualization [55]. Persons who perceive goodness in this manner are thereby consciously engaged in a self-reflective process of character development whereby good and bad actions are seen as habit-forming. This is the singular defining aspect of the virtue-theoretical point of view that must be identified for any scale to isolate it as its own distinct frame, and is what our PMFM tool uniquely measures.

### Constructing and validating the Philosophical Moral-Framing Measure (PMFM)

In four settings where adult subjects were given the option of acting altruistically—by sacrificing either class credit, money, or physical energy and time—we administered our 12-question Philosophical Moral Framing Measure (PMFM) on a 5-point Likert scale from “strongly agree” to “strongly disagree,” We designed this measure to distinguish philosophical heuristics associated or disassociated with altruistic behavior. We relied on the extant philosophical and experimental literature’s four canonical moral frames as construed above, namely deontology, utilitarianism, virtue theory, and egoism as guideposts for scale design. In this way, we used a deductive item-generation approach based on a set of theories which represent different moral frames in use, instead of a more inductive method which may have resulted in a broader set of initial questions. In other words, based on extant theory, we expect our set of items to fit into predetermined moral frames, although previous literature does not make clear whether people generally interpret moral issues as fitting these distinct frames. This approach allowed us to reduce response bias, especially that caused by fatigue [82]. In all, our measure contained 12 items meant to capture four subscales as described below.

#### Deontology

We measured subjects’ tendency to reason along broad deontological lines with the following two questions designed to assess the extent to which subjects embraced (1) the concept of moral duty, (2) a commitment to logic, (3) non-consequentialist values, and (4) an aversion to exploiting others. We also sought indirect negative confirmation via independence from the other four frames.

- I try to never break any moral rules.
- I try to think and act logically in every situation.
- A good intention is more important than a good result.
- I think no one should have to suffer for the benefit of others.

#### Utilitarianism

We measured subjects’ tendency to reason along broad utilitarian lines with the following three items designed to assess the extent to which subjects (1) embraced a commitment to maximizing the good for the greatest number, (2) a preference for feelings over logic, (3) consequentialist thinking, and (4) a preference for results over rules. We also sought indirect negative confirmation via independence from the other four frames.

- I try to do whatever brings the most happiness for the most people.
- It matters more to feel good than to think and act logically.
- The results of my actions matter more than why or how I go about doing them.
- I sometimes break a moral rule if doing so will achieve the best result.

#### Virtue theory

We measured subjects’ tendency to reason along traditional virtue-theoretical lines with the following two questions in the PMFM questionnaire designed to assess the extent to which subjects embraced (1) an aspirational moral self-image, (2) a conception of ethics as self-actualizing, and (3) an aversion to excess. We also sought indirect negative confirmation via independence from the other four frames.

- When I choose to act ethically, I am also choosing to become a better person.
- Acting ethically is more personally fulfilling to me than acting unethically.
- Too much of anything is bad.

#### Egoism

We measured subjects’ tendency to reason along standard egoistic lines with a single unambiguous question designed to measure the extent to which subjects embraced a fundamentally self-interested attitude. We also sought indirect negative confirmation via independence from the other four frames, while recognizing the possibility for some potential overlap with the three consequentially-oriented questions in the utilitarian frame given that both frames have this aspect in common.

- I tend to place my own interests above those of others.

## Studies

This study was approved by the Fordham University Institutional Review Board and all data were analysed anonymously. A written consent for was provided in all studies and no study included minors.

As Hinkin [82] states, in order to demonstrate construct validity, a link must be formed between theory and measurement such that the operationalization of a set of items displays content validity, criterion-related validity, and internal consistency. To establish content validity, we expose our theoretically developed item bank to a principal component analysis in Study 1 in line with the recommendations of Ford and colleagues [83]. This allows us to assess whether persons interpret moral frames as indicated in the philosophy literature, and whether we missed any categorizations in our initial assessment. Briefly, this analysis—and that of subsequent studies—indicates that we missed one potential moral frame in our initial accounting: self-abnegation. In Study 2, we assess the goodness of fit of the underlying factor structure using confirmatory factor analysis from an independent sample. Finally, in Study 3, we replicate these findings in a field setting to assess both the external and ecological validity of our measure.

In all studies, we assessed internal consistency by using Cronbach’s Alpha [82,84] with the suggested cutoff of .70 for exploratory measures [85]. Additionally, we assessed criterion validity by relating our measure to an altruistic outcome, giving credit, money, or physical energy and time to a cause or person.

### Study 1

#### Participants

Three hundred and fifteen undergraduate students completed our initial reliability and validity test for five extra credit points towards an exam in their organizational behavior course. These participants were recruited through a one-time email from the total set of introductory organizational behavior courses taught at a midsize college in the northeastern United States. No exclusion criteria were applied to our recruitment besides enrollment in introduction to organizational behavior. Because our sample comprised college students, the results of Study 1 cannot be reliably applied to an adult population or a population involving non-college educated individuals. The average age of our sample was 20.25 years (*SD* = 2.41); 53% were male, 71% were White, 19% were Asian, 7% were Hispanic and 3% were Black.

#### Procedure

Participants entered the lab and completed a written informed consent. Then, all participants completed the 12-item PMFM in the lab on the Qualtrics survey platform. Surveys were given online in order to randomize the order of the items. After completing the PMFM, on a separate page, participants were told “due to illness a few students have not been able to participate fully in class over the last few weeks. We are giving you the opportunity to donate a portion of the extra credit you will earn from participating in this study to one of these students. Would you be willing to donate a portion of your extra credit to one of your classmates?” If participants indicated they would, they were then asked what percentage (0–100) they would be willing to donate. This was used as a measure of altruistic behavior because participants were given the opportunity to anonymously sacrifice a portion their own class credit for the benefit of an unidentified classmate. Such an action would appear to be intrinsically altruistic, since the gifting was blind, private, and anonymous, thus not bestowing instrumental advantage through increased social standing. Furthermore, the fact that participants were required to decide immediately after filling out the ethical questionnaire whether to give and how much, increased the likelihood that they exercised a degree of moral reflection on their decision. After indicating whether they would donate a portion of their extra credit students were debriefed and excused from the lab.

#### Results

*Principal components analysis*. As an initial test of the underlying structure of our measure, we first conducted a principal components analysis with a promax rotation to determine if the survey responses matched the theoretical dimensionality of the scale [82]. In other words, we attempted to determine if students were able to differentiate the four theoretically distinct dimensions of the PMFM. As expected, four components displayed an Eigenvalue over 1. These factors explained 63.55% of the variance in the items—which crossed the 60% minimum recommended threshold [82]—and each was greater than the comparison eigenvalues [86]. In addition, a scree plot showed that these four factors settled before its elbow, while the others settled after it [86].

To analyze factor loadings, Hinkin [82] recommends a cut-off of .40. Alternatively, Tabachnick and Fidell [87] endorse a cutoff of .30 since this value represents less than 10% shared variance between the factors. We chose this less conservative cutoff to allow a greater opportunity to identify cross-loadings between the factors. Thus, we ignored factor loadings of less than .30 (e.g. [88]). Our results indicated that while our sample was generally able to differentiate the theoretical frames underlying social behavior, there were a few differences. The promax pattern matrix can be seen in Table 1. Mainly, items 1, 2, 9, and 10 loaded onto a first factor that accounted for 21.93% of the variance observed. Theoretically, items 1 and 2 represent deontology and items 9 and 10 represent virtue theory. Thus, it appears that the study participants recognized a combined deontology-virtue factor. While this association was unforeseen, it is understandable given that the two frames are not necessarily in mutual contradiction. This association is significant and suggests an avenue for further research into the extent to which they are, in fact, conjoined.

**Table 1:** Study 1—Exploratory factor analysis pattern matrix.

Item	Component			
	1	2	3	4
1. I try to never break any moral rules.	0.757			
2. I try to think and act logically in every situation.	0.809			
3. I think no one should ever have to suffer for the benefit of others.				-0.878
4. A good intention is more important than a good result.			0.837	
5. I try to do whatever brings the most happiness for the most people.			0.767	
6. The results of my actions matter more than why or how I go about doing them.		0.848		
7. It matters more to feel good than to think and act logically.			0.826	
8. I sometimes break a moral rule if doing so will achieve the best result.		0.779		
9. When I choose to act ethically, I am also choosing to become a better person.	0.824			
10. Acting ethically is more personally fulfilling to me than acting unethically.	0.798			
11. Too much of anything is bad.				0.891
12. I tend to place my own interests before those of others.		0.828		

Principal Component Analysis. 4 components extracted.

A second factor accounting for 15.11% of the variance observed contained items 12, 6, and 8. Taken together, the items were all theorized to represent egoism; thus we can assert that a clear egoism factor emerged from the data. Items 4, 5 and 7 loaded onto a third factor that accounted for 14.60% of the variance observed. These items indicated a utilitarian frame also was present in the data. Finally, a fourth factor accounting for 11.90% of the variance observed contained items 11 and 3, and item 3 weighted negatively on it. These two items were (3) “Too much of anything is bad,” intended to represent virtue theory, and (11) “I think no one should have to suffer for the benefit of others” intended to positively represent deontology. However, since item 3 weighted negatively on this factor, it could be said to be made up of the following two considerations: too much of anything is bad; and, someone *should* have to suffer for the benefit of others. We did not anticipate this fourth factor based on our review of the philosophical literature, however, based on our empirical analysis we can state that our participants recognized its existence. In our view, taken together, this fourth factor may indicate a self-abnegation moral frame that rejects hedonism (3) while embracing suffering for the benefit of others (11) and suggests an avenue for further research. Additionally, no items loaded on multiple factors, indicating that each item was interpreted as belonging to a single moral frame.

*Reliability*. Following the factor analyses, we calculated Cronbach’s alpha values for all four subscales of the PMFM. Each passed the .70 threshold [85]. Specifically, the Cronbach’s alpha of the first factor (deontology-virtue) was .81, the Cronbach’s alpha of the second factor (egoism) was .75, the Cronbach’s alpha of the third factor (utilitarianism) was .74, and the Cronbach’s alpha of the fourth factor (self-abnegation) was .73, providing support for the reliability of our subscales.

*Validity in predicting altruism*. After testing the reliability of the PMFM, we calculated means, standard deviations, and correlations between the PMFM factors, demographic variables, and donation behavior. This can be seen in Table 2. Respective items were average to create subscale values (e.g. the deontology-virtue subscale is the mean of participant responses to items 1, 2, 9, and 10).

**Table 2:** Study 1—Correlations and descriptive statistics.

Variable	Mean	SD	1	2	3	4	5	6
1. Deontology-Virtue	5.51	0.92	----					
2. Egoism	4.18	1.14	-0.16**	----				
3. Utilitarianism	4.58	1.03	0.07	-0.06	----			
4. Self-Abnegation	4.47	1.50	-0.02	0.08	0.03	----		
5. Age	20.16	2.41	-0.07	-0.04	0.01	0.03	----	
6. Sex (Male = 1, Female = 0)	0.53	----	-0.10	0.19**	-0.10	0.06	-0.03	----
7. Donation Percentage (0–100%)	12.82	24.73	0.21**	-0.23**	0.13*	0.01	0.03	0.04

*p < .05

**p < .01

As stated, we gave participants the opportunity to donate a chosen portion of their extra credit to a needy student. Since neither the participant nor the needy student were identified, no instrumental benefit could be derived via reciprocation or increased social standing, thereby making the act intrinsically altruistic. We ran a series of four binary logistic regressions, regressing whether students were willing to donate to their needy peer (0 = no; 1 = yes) onto each individual subscale, to explore whether any individual factor increased or decreased a participant’s likelihood to donate his or her extra credit. We found that deontology-virtue (*B* = .37, *SE* = .14, *p* < .01, *Exp(B)* = 1.45) and utilitarianism (*B* = .25, *SE* = .12, *p* = .04, *Exp(B)* = 1.28) did increase the likelihood, while self-abnegation did not (*B* = −.01, *SE* = .08, *p* = .91). In addition, egoism negatively impacted a participant’s willingness to donate (*B* = −.41, *SE* = .11, *p* < .01, *Exp(B)* = .66). Then, we simultaneously regressed whether students were willing to donate to their needy peer onto all four subscales. This binary logistic regression indicated that only deontology-virtue (*B* = .27, *SE* = .14, *p* = .05, *Exp(B)* = 1.31) and egoism (*B* = −.37, *SE* = .11, *p* < .01, *Exp(B)* = .69) significantly predicted willingness and unwillingness to donate, again, when all four subscales are considered simultaneously.

Then, we conducted a series of four linear regressions, regressing the amount of credit (0–100%) students agreed to donate to their needy peer onto each individual subscale. Individually, deontology-virtue (β = .21, *t* = 3.87, *p* < .01) and utilitarianism (β = .13, *t* = 2.33, *p* = .02) increased the amount of the donation, while egoism decreased it (β = −.23, *t* = −4.25, *p* < .01). Again, self-abnegation had no effect (β = .01, *t* = .01, *p* = .99). This time, however, when considered jointly (e.g. when we simultaneously regressed the amount of credit students donated to their needy peer onto all four subscales), deontology virtue (β = .17, *t* = 3.11, *p* < .01), utilitarianism (β = .11, *t* = 1.99, *p* = .05), and egoism (β = −4.36, *t* = −3.65, *p* < .01) all impacted donation amount. Finally, we replicated this regression analysis considering only those people who chose to donate (n = 116). This analysis indicated that only deontology-virtue predicted donation amount (β = .26, *t* = 2.66, *p* < .01) in this subsample.

#### Discussion

These results strongly suggest that moral framing is predictive of adult altruistic behavior, depending on the moral frame employed. Specifically, egoistic moral framing is strongly predictive of decreased altruism, while deontology-virtue framing is strongly predictive of increased altruism. There has been considerable deontic justice extrapolation of altruistic behavior in dictator-game findings [1–2,6]. Though our model does not include a justice component *per* se, our results compliment these extrapolations, for this sample affirmed a commitment to (1) following moral rules as well as (2) thinking and acting logically in every situation. These are strong deontic indicators, revealing adherence to rational moral principles as opposed to felt needs, which is how altruistic behavior is often interpreted [29]. However, the utilitarian group did register emotive sensitivity (4, 5, 7) which was also predictive of increased altruism, but to a lesser extent when distinguished from deontology-virtue. Furthermore, the virtue-theoretical associations (9, 10) confirm that such behavior may be motivated by an aspirational moral self-image [28]. Thus, our results indicate that deontological framing and aspirational moral self-image (virtue) may be underlying altruistic behavior, while egoistic moral framing is strongly inhibiting.

### Study 2

Study 1 provided evidence that our scale fit a logical and theoretically sound factor structure. We conducted Study 2 to verify this factor structure by conducting a confirmatory factor analysis to examine whether our hypothesized relationship—between our items and their underlying latent constructs—exists. To accomplish this, in Study 2 we replicated Study 1 with our new dependent measure–donating money to charity. This was used as a measure of altruistic behavior because participants were given the opportunity to anonymously sacrifice a portion their earnings for the benefit of an unknown needy person. Such an action would appear to be intrinsically altruistic, since the gifting was blind, private, and anonymous, thus not bestowing instrumental advantage through increased social standing. Furthermore, the fact that participants were required to decide immediately after filling out the ethical questionnaire whether to give and how much, again increased the likelihood that they exercised a degree of moral reflection on their decision.

#### Participants

Three hundred and seventeen working adults from within the U.S. participated in Study 2. Their mean age was 30.43 years (*SD* = 10.35); 66% were male, 74% were White, 13% were Asian, 7% were Black and 5% were Hispanic; 1% did not indicate race. These participants were recruited via Amazon Mechanical Turk and were paid $1.00 for their participation. We limited our sample to adults in the United States that had at least a 95% response rate in previous Mechanical Turk tasks and had completed at least 500 previous tasks. Validation studies have shown that Mechanical Turk can strongly reduce the potential for non-response error in online research [89] and that results obtained through the service do not substantially differ from those obtained through university subject pools [89], such as Study 1 above.

#### Procedure

As in Study 1, after completing a written electronic consent form, participants completed the 12-item PMFM. Then, on a separate page, because Mechanical Turk respondents receive financial compensation instead of the option of donating extra credit, respondents were given the option to donate a percentage of their remuneration (0%–100%) to those in need during the holidays. Specifically, since our participants were recruited and data were collected in December, participants were told, “Finally, every year certain families cannot afford basic necessities around the holidays. Since it is the holiday season, we are giving you the opportunity to donate a portion of the pay you will earn from participating in this study to those in need. Would you be willing to donate a portion of your pay?”

#### Results

*Confirmatory factor structure*. First, we tested the factor structure of the PMFM using IBM SPSS AMOS 24.0. We chose maximum likelihood estimation because our data were normally distributed. Means, standard deviations, and correlations between the PMFM factors, demographic variables, and donation behavior can be seen in Table 3. We hypothesized a four-factor model, based on the results of our exploratory factor analysis, conducted in Study 1. The result of a CFA including our four extracted factors (deontology-virtue, egoism, utilitarianism, self-abnegation) indicated that the data fit this model well, (*CFI* = .97, *CMIN/DF* = 1.68, *RMSEA* = .05, *SRMR* = .11) and better than a one factor model (*CFI* = .52, *CMIN/DF* = 10.72, *RMSEA* = .18, *SRMR* = .31, χ^2^(6) = 496.61, *p* < .01).

**Table 3:** Study 2—Correlations and descriptive statistics.

Variable	Mean	SD	1	2	3	4	5	6
1. Deontology-Virtue	5.38	0.99	----					
2. Egoism	4.06	1.12	-0.32**	----				
3. Utilitarianism	4.25	1.15	0.11*	0.18**	----			
4. Self-Abnegation	4.13	1.43	0.09	-0.09	-0.11*	----		
5. Age	30.43	10.35	0.13*	-0.14*	-0.17**	0.02	----	
6. Sex (Male = 1, Female = 0)	0.66	----	-0.15**	0.20**	-0.04	-0.11	-0.23**	----
7. Donation Percentage (0–100%)	12.98	26.38	0.18**	-0.23**	0.11	-0.04	0.09	-0.06

*p < .05

**p < .01

*Reliability*. Again, we computed Cronbach’s alpha values for all the subscales. All were above the .70 threshold (deontology-virtue = .83; egoism = .73, utilitarianism = .76) except self-abnegation (.66).

*Validity in predicting altruism*. As in Study 1, since neither the participant nor the needy student was identified, no instrumental benefit could be derived via reciprocation or increased social standing, thereby making the act intrinsically altruistic. A series of four binary logistic regressions, regressing whether participants were willing to donate a portion of their earnings (0 = no; 1 = yes) onto each individual subscale, indicated that individually, deontology-virtue (*B* = .65, *SE* = .15, *p* < .01, *Exp(B)* = 1.92), utilitarianism (*B* = .24, *SE* = .11, *p* = .03, *Exp(B)* = 1.27), and self-abnegation (*B* = .20, *SE* = .08, *p* = .02, *Exp(B)* = 1.22) all increased participants’ likelihood of donating part of their winnings, while egoism (*B* = −.41, *SE* = .11, *p* < .01, *Exp(B)* = .66) decreased it. As in Study 1, we then regressed whether participants were willing to donate a portion of their earnings simultaneously onto all subscales. When considered together, all four factors continued to account for unique variance in the probability of donating part of one’s winnings (deontology-virtue, *B* = .46, *SE* = .16, *p* < .01, *Exp(B)* = 1.58; utilitarianism, *B* = .32, *SE* = .12, *p* < .01, *Exp(B)* = 1.37; self-abnegation, *B* = .18, *SE* = .09, *p* = .04, *Exp(B)* = 1.20; egoism, *B* = −.35, *SE* = .12, *p* < .01, *Exp(B)* = .71).

Then, we explore how these factors impacted the amount participants donated. A series of four linear regressions, regressing the amount donated by participants onto our four individual factors, indicated that only deontology-virtue (β = .18, *t* = 3.20, *p* < .01) and egoism (β = −.23, *t* = −4.13, *p* < .01) significantly impacted donation amount in the expected directions. Utilitarianism (β = .11, *t* = 1.87, *p* = .06) and self-abnegation (β = −.04, *t* = −.72, *p* = .47) did not. However, when we conducted an additional linear regression where donation amount was simultaneously regressed on all four factors, only egoism (β = −.22, *t* = −3.79, *p* < .01) and utilitarianism (β = .13, *t* = 2.25, *p* = .03) significantly predicted it. Finally, when looking at only those participants who donated (n = 108), only egoism impacted donation amount (β = −.21, *t* = −2.25, *p* = .03) and did so negatively.

#### Discussion

Study 2 confirmed the factor structure of the PMMFM identified in Study 1. Again, we found four factors underlying it including the existence of a combined deontology-virtue factor and a self-abnegation factor. Contrary to Study 1, which found that only deontology-virtue an egoism impacted donation likelihood, in Study 2 we found that deontology-virtue, utilitarian, and self-abnegation frames all positively related to participant’s likelihood of donating some of their earnings. However, we did find that only deontology-virtue and egoism related to the amount donated.

### Study 3

Study 3 had two purposes. First, we attempted to put our scale to a conservative test and recruit only a sample of people who might be more altruistically inclined. To do this, we surveyed people participating in the New York City annual March of Dimes and A.I.D.S. Walk. Second, this setting allowed us to validate our measure in a field setting centered on social morality. It is important to note, however, that since these walks could be construed as high-profile social events for certain employers or other groupings, some of the participants may well have been instrumentally inspired to participate, and thus may not necessarily have been acting purely out of altruism.

#### Participants

Three hundred and eighty-three adults participated in Study 3; the mean age of respondents was 31.06 years (*SD* = 12.10). In addition, 33% were male, 30% were White, 29% were Black, 28% were Hispanic and 13% were Asian. Thus, this sample was much more racially diverse than our previous ones. These participants were recruited from two subsamples involving walkers at the 2016 New York City March of Dimes and A.I.D.S. Walk. 127 participants were recruited from the A.I.D.S. walk, while 256 were recruited from the March of Dimes. The two subsamples were similar demographically. The mean age of respondents from the March of Dimes was 30.33 years (*SD* = 12.04) 30% were male, 31% were White, 27%% were Black, 16% were Asian, and 26% were Hispanic. The mean age of respondents from the A.I.D.S. Walk was 32.56 years (*SD* = 12.14); 39% were male, 27% were White, 35% were Black, 6% were Asian, and 32% were Hispanic.

#### Procedure

Eight research assistants approached individuals at the March of Dimes who had gathered at the staging area before the walk began. Similarly, three research assistants approached walkers in the staging area of the A.I.D.S. walk. These research assistants asked walkers if they would complete a paper and pencil survey to help researchers from a local university. If walkers agreed to participate, they filled out a written consent form. We did not use any exclusion criteria to eliminate potential participants.

After completing the consent form, participants completed the 12-item PMFM. Finally, on a separate page, we asked participants 1) how many people had donated to their effort and 2) how much money they had raised from those individuals. Because the two walks draw similarly inclined individuals under the premise of raising money for a given cause, and in order to ensure sufficient data for our items, we combined the two datasets for our analysis.

#### Results

*Confirmatory factor analysis*. In this case, we wanted to examine the ecological validity of our measure by replicating by the CFA conducting in Study 2 in a field setting. The confirmatory model mirroring that in Study 2 fit the data well (*CFI* = .93, *CMIN/DF* = 3.02, *RMSEA* = .07), significantly better than a single factor model (*CFI* = .63, *CMIN/DF* = 10.18, *RMSEA* = .16, χ^2^(6) = 404.67, *p* < .01). We were unable to calculate SMRM for this data set due to missing values.

*Reliability*. The Cronbach’s alpha of our deontology-virtue measure was .84. The Cronbach’s alpha of our egoism measure (items 2, 3, 13) was .72. The Cronbach’s alpha of our utilitarianism measure was .71, and the Cronbach’s alpha of our self-abnegation measure was .32. Thus, as in Study 2, all scales were reliable except self-abnegation.

*Validity in predicting altruism*. Means, standard deviations, and correlations among Study 3 variables can be seen in Table 4. We conducted eight linear regressions to test whether the PMFM was able to predict altruistic behavior. In the first four, we regressed the number of sponsors each individual recruited to support him or her onto each dimension of the PMFM. In the second four, we regressed the amount each walker raised onto each subscale of the PMFM. In this sample, individually, deontology-virtue predicted the number of sponsors the individual walk participant had recruited as support for his or her walking effort (β = .16, *t* = 2.50, *p* = .01) and funding amount those sponsors pledged to donate (β = .24, *t* = 3.85, *p* < .01). The same went for utilitarianism (sponsors: β = .15, *t* = 2.28, *p* = .02; funding amount: β = .19, *t* = 3.04, *p* < .01). Interestingly, in this context, egoism also positively related to number of sponsors (β = .16, *t* = 2.55, *p* = .01) and funding amount (β = .22, *t* = 3.56, *p* < .01). Further, when we regressed number of sponsors onto the subscales of the PMFM, egoism was solely responsible for predicting it (β = .13, *t* = 1.94, *p* = .05). When funding amount was regressed on these subscales, egoism remained a significant predictor of it (β = .17, *t* = 2.62, *p* < .01) as did deontology-virtue (β = .19, *t* = 2.77, *p* < .01). Utilitarianism did not (β = .05, *t* = .70, *p* = .49), however.

**Table 4:** Study 3—Correlations and descriptive statistics.

Variable	Mean	SD	1	2	3	4	5	6	7
1. Deontology-Virtue	5.73	1.12	----						
2. Egoism	4.31	1.37	0.14*	----					
3. Utilitarianism	5.06	1.28	0.38**	0.28**	----				
4. Self-Abnegation	3.71	1.13	-0.10	0.03	-0.19**	----			
5. Age	31.01	12.10	0.18*	0.01	-0.02	0.03	----		
6. Sex (Male = 1, Female = 0)	0.33	----	0.01	-0.08	-0.03	0.05	0.01	----	
7. Number of Sponsors	10.83	13.79	0.16*	0.16*	0.15*	0.01	0.29**	-0.06	----
8. Money Raised (US$)	737.60	1160.96	0.24**	0.22**	0.19**	-0.02	0.24**	-0.11	0.53**

*p < .05

**p < .01

#### Discussion

Overall, Study 3 replicated the PMFM factor structure identified in Study 1 and confirmed in Study 2, and did so in an environment where many people were likely more similar in their moral framing than the general population. It also confirmed that moral frames relate to altruistic-type behavior. However, one difference was found between Study 3 and our previous two studies. Mainly, egoism positively related to both number of sponsors and funding amount. This result is consistent with our other findings, while intimating that in a public context, those who are instrumentally-motivated by the activity want to stand out by raising more funds from more people. Indeed, social status being a possible motivator in this study, although all participants were certainly acting prosocially, they were not necessarily behaving completely—or even genuinely—altruistically. Some actually could have been primarily instrumentally motivated given the context.

## General discussion

The motivation for our study was our observance of scholars extrapolating philosophical framing from observed altruistic behaviors, but only relying on relatively few traditional frames in so doing, namely, deontic justice in organizational behavior and virtue theory in law and economics. Given that many competing moral frames can manifest as identical behavior, we saw a need for a more rigorous method for discerning the actual motivations of study subjects [7]. Our study offers just such an instrument—the Philosophical Moral-Framing Measure (PMFM), which we recommend be deployed when altruistic behavior is observed by researchers. As the factor loadings drawn from data in three different environments clearly show, adults recognize heuristic forms of competing philosophical moral frames, namely, deontology-virtue, utilitarianism, and egoism, and these frames significantly underlie altruistic motivation or its opposite. Our research also provides mixed support that people may recognize self-abnegation as playing a role. These findings hold the potential to inform ongoing conversations regarding organizational citizenship [90–91], and moral crowding out [4]. We address these implications in the Practical Implications section below.

It is important to note that the PMFM is not meant as a measure of philosophical expertise, as we do not presume that ordinary subjects have any such training. Nevertheless, heuristic approximations of traditional philosophical frames may be internalized to some extent in the wider culture. Therefore, the PMFM is rather meant as a measure of interpreted moral frames. For instance, an average adult may well construe his or her actions virtue-ethically by recognizing them as self-actualizing, yet ignore other aspects of the virtue-theoretical frame such as the importance of moderation. Similarly, that same adult may at the same time adhere to deontic heuristics of logical consistency and strict adherence to moral principle yet overlook more nuanced implications of this frame such as that no one should be made to suffer for the benefit of others. A number of such instances did in fact arise during validation and are inevitable given the complexity of the theories and that even philosophers will at times disagree about the norms implicit within each frame. These are discussed in the following section.

### Surprise findings

In each of our studies, we found that deontology and virtue theory were always conjoined in practice This finding is not necessarily at odds with previous literature since neither literature explicitly restricts itself to a single moral frame, and since deontology and virtue theory are not obviously contradictory [92], this is a significant finding linking the two constructs. However, it does suggest that deontic extrapolations of observed altruism in dictator game studies in the organizational literature may be accurate yet not fully explanatory. For aspirational motives of self-actualization may also be at work in such instances. Conversely, the virtue theoretical motivations underlying civic engagement in the law and economics literature may also be influenced by a deontic commitment to logical moral principle. More research is needed to determine the extent to which these two frames are conjoined in ordinary, if not philosophical, practice.

Regarding utilitarianism and egoism, we had originally expected some overlap with the three purely consequentialist utilitarian questions given that both frames have this aspect in common. Thus, if any of these loaded with “I tend to place my own interests above those of others,” this would be strongly indicative of an egoistic consequentialism. But if answered in the negative—signaling a rejection of egoistic behavior and linked with any of the consequentialist questions, this would reflect a utilitarian consequentialism. What we found was that two out of three consequentialist questions always loaded positively with egoism, attesting to a classic egoistic outlook that (1) preferences personal interests and (2) is willing to flout moral rules and (3) overlook justifications to satisfy them. This resulted in providing us with a robust frame-specific indicator for egoism. Table 5 distinguishes our theoretical and empirical item categorizations.

**Table 5:** Theoretical and empirical PMFM item categorizations.

Item	Theoretical Categorization	Empirical Categorization
1. I try to never break any moral rules.	Deontology	Deontology-Virtue
2. I try to think and act logically in every situation.	Deontology	Deontology-Virtue
3. I think no one should ever have to suffer for the benefit of others.	Deontology	Self-Abnegation (Reversed)
4. A good intention is more important than a good result.	Deontology	Utilitarianism
5. I try to do whatever brings the most happiness for the most people.	Utilitarianism	Utilitarianism
6. The results of my actions matter more than why or how I go about doing them.	Utilitarianism	Egoism
7. It matters more to feel good than to think and act logically.	Utilitarianism	Utilitarianism
8. I sometimes break a moral rule if doing so will achieve the best result.	Utilitarianism	Egoism
9. When I choose to act ethically, I am also choosing to become a better person.	Virtue Theory	Deontology-Virtue
10. Acting ethically is more personally fulfilling to me than acting unethically.	Virtue Theory	Deontology-Virtue
11. Too much of anything is bad.	Virtue Theory	Self-Abnegation
12. I tend to place my own interests before those of others.	Egoism	Egoism

On a related point, our findings suggest that egoists may be significantly more consequentially-motivated than utilitarians. For we had originally conceived the question “a good intention is more important than a good result” as a rejection of consequentialism signaling deontology. However, since it loaded strongly and systematically with two clearly utilitarian norms (1) achieving the greatest happiness for the greatest number and (2) preferencing feelings over logic, it appears that subjects more likely interpreted the question as underscoring optimific goal-directedness rather than rejecting consequentialism. There is also some debate as to whether utilitarianism necessarily requires discounting intentions to begin with as doing so would seem to contradict an essential component of moral development [93]. As Piaget points out, children learn at a young age that mistakes happen even from the best intentions, and that it would be wrong not to praise the aim of a good deed even under unforeseen negative consequences. A utilitarian would thus arguably praise the action as optimifically goal-directed [94], while other frames may be more ambivalent on such matters since none recognize an obligation to maximize the happiness of others as first principle.

A fourth unexpected finding was that egoism and deontology-virtue were equally high predictors of altruism in the public context of study 3 where social status was a possible motivator. This finding suggests that egoists were cynically or relationally motivated by their own instrumental interests instead of those of others. Further research might indicate that persons exhibiting socially aversive ‘dark triad’ personality traits of Machiavellianism, narcissism, and psychopathy [95] might also exhibit high levels of seemingly-altruistic behavior in public contexts for entirely self-serving reasons.

Finally, we designed our instrument to only measure the aforementioned Big 4 philosophical frames, yet were surprised to find a fifth also emerge from the data, which we decided to call “self-abnegation.” It appeared to be a tendency to reject hedonistic excess (a) “too much of anything is bad”—a question originally conceived to measure the guiding virtue-ethical ideal of moderation but failed to do so in practice—while also embracing the idea that (b) some persons “should have to suffer for the benefit of others”—a question originally conceived to measure deontological injustice that failed to do so in practice. We had not anticipated that any subjects would recognize a moral obligation toward suffering and self-denial, nor that this norm would indicate a new moral frame supporting it. We initially wondered if this frame might be restricted to the context of the Jesuit university setting in which our first study was administered. However, we found continued evidence of this factor loading in Study 2 in an entirely different and secular setting, therefore suggesting that Catholicism may not have played a significant role. Its emergence as a meaningful factor across samples suggests the existence of a distinct moral frame defined by a degree of self-denial. This finding may lend further support to existing behavioral research on the role of self-control in resisting enticement [96], though self-control may also be at work in each of the other frames depending on the context. Interestingly, self-abnegation failed to appear in Study 3. One explanation could be that the self-congratulatory social context of benefit walks had an inhibiting effect on anti-hedonistic norms. Still, based on our findings, we suggest more research be done on the recognition of a self-abnegation frame, especially since it clearly appeared in two of our three studies. In particular, it would be interesting to investigate a possible connection with recent evidence suggesting that lay respondents preferring utilitarianism in sacrificial dilemmas will oppose impartial beneficence and instrumental harm [54]. As the authors point out, the embrace of instrumental harm has been associated with subclinical psychopathy. Therefore, the norm could be influenced by egoism. However, it could also be influenced by a norm of self-abnegation, a value that may also be consistent with certain forms of utilitarianism such as rule utility and preference utility. Sorting out these possible conflicting implications would be illuminating.

#### Limitations

While our studies help to establish broad differences between philosophical moral frames and initially test how laypersons ordinarily acknowledge these frames, they do have some limitations. First, we cannot state with certainty that subjects applied abstract philosophical concepts justifying their actions. Instead, a variety of psychological mechanisms motivate people to make decisions that theorists can then describe as happening to be consistent with abstract principles. Still, given that these frames are widespread in the culture and educational literature, it is reasonable to take their predictive effect on altruistic behavior as potentially cognitively and motivationally significant.

Second, we cannot state with certainty that all participants in the two walks to raise money for their respective causes were doing so out of altruistic reasons in Study 3. We need to recognize that firms often make requests of employees to participate in such events in order to strengthen group bonds through a common experience. Thus, it is challenging to distinguish whether the individual was participating out of truly altruistic reasons, or whether s/he was doing so for social or reputational reasons. Nevertheless, the instrument did have sufficient power to distinguish these four major frames, whether or not those driven by non-altruistic reasons were present. Additionally, this provides insight into several practical applications of our findings, discussed below. The instrument also could capture individuals so motivated if their own moral frames were aligned—those individuals would load onto the egoism frame, which would capture instrumental and relational self-interest [14,23].

Third, we recognize that there are numerous moral frames or theoretical subcategories under each of the four frames that we examined that we could have measured and distinguished. This includes, notably, the feminist ethics of care approach [97], which falls broadly within the scope of virtue theory and various act-versus-rule-utilitarian approaches that fall within the overarching utilitarian frame. Furthermore, our instrument does not seek to measure justice framing *per* se, which is a subcategory within the Big 4 philosophical frames. Given that the instrument was intended to be used alongside an altruistic manipulation (i.e., in which experimental subjects faced the choice of making a tangible personal sacrifice), we believed that our instrument needed to be limited in length, yet informative in discernment. A high number of scale items could quickly make the instrument unwieldy, increasing burdens on subjects and researchers, and possibly undermining the attention subjects might place on the instrument. We recognize the value of these other frames or subcategories and welcome researchers to explore how to distinguish them as parsimoniously yet rigorously as possible in future research endeavors.

Fourth, we recognize that individuals may have responded to the survey by providing answers that they believe we as researchers were hoping to hear. Social desirability bias is well documented in survey-based or experimental scholarship [98]. Luckily, our research draws from anonymous paper and online surveys, the latter implemented through Amazon Mechanical Turk. Research [99] shows that individuals report lower social anxiety and social desirability when responding anonymously (than when non-anonymously), and when using the Internet (than when using paper-based methods).

Fifth, although our three studies included three different samples (college students, working adults in the United States, and charity walkers), Studies 1 and 3 employed convenience samples and all studies employed samples taken from within the United States. As such, all samples include participants from a rich, industrialized, democratic country. Notably, in Study 1 our convenience sample included college age students in the northeastern United States. These students have been educated on certain ethical principles and most come from middle or upper-class backgrounds. Because convenience sampling is a non-probability sampling technique and because these students come from a limited age range, and socioeconomic status has been linked to ethical beliefs and behavior [100] including prosocial behavior [30] and empathy [101], this sample may not representative of the broader global population. Again, while we try to account for this possibility in Studies 2 and 3, all samples are Western and Western ethical thinking has been shown to differ from that found in Eastern parts of the world [102]. Therefore, while consistent, our results should be considered in the context of ethical framing within the United States, possibly expanding to other Western industrialized countries.

Finally, we did not measure discriminant validity in any of our studies by directly comparing the PMFM to other validated measures of moral framing. We made this decision because our main purpose was to validate items representing the four frames described as they relate specifically to seemingly altruistic behavior. Still, new validated scales exist that measure specific components of our model, such as Kahane and Colleagues new Oxford Utilitarianism scale [54] and Piazza and Sousa’s Consequentialist Thinking Scale [103]. We recognize the value of these measures and welcome researchers to explore how to distinguish them from our own as parsimoniously yet rigorously as possible in future work.

### Practical implications

The findings of our three studies broadly confirm what previous extrapolations have suggested—that seemingly altruistic behavior is associated with moral frames internalized within the individual. Practically, however, our research shows that moral frames may present in more nuanced manners than prior work has suspected. Specifically, we find clear evidence that deontological and virtue-theoretical norms are intertwined. This is a highly significant finding that helps to connect the two existing moral framing literatures informed by these respective philosophical approaches. Given our results, the deontic framing extrapolated from organizational settings including dictator-game scenarios may well be motivated as much by aspirational virtue-theoretical concerns linked to moral self-image [28]. This seems to make intuitive sense considering that since deontology is not goal-directed, it provides precious little material incentive to act ethically. Virtue theory however provides an intrinsically satisfying positive self-image—an aspect explicitly appealed to in the PMFM questions measuring the virtue-theoretical frame, suggesting that ethical actions are both intrinsically fulfilling and aiming to make the agent a better person. This may provide a kind of psychological payoff to deontically-oriented persons. If confirmed, this would be a highly significant behavioral finding, indicating that moral priding can function as a particularly effective motivational tool for such individuals. Extant literature on moral crowding out has shown that instrumental appeals tend to demotivate those with medium or high intrinsic motives [4]. Our findings thereby suggest an alternate motivational avenue for such persons—one directed at aims of deontology-virtue.

Conversely, utility-oriented persons may be more moved by empathic concern for the general wellbeing of all. And as the results of Study 2 show, utilitarian framing does seem to be at work in the thinking of some altruistically-inclined individuals. It would hence be interesting to use the PMFM in the context of dictator-game and other altruistic research contexts to see to what extent the frames measured are at work in the moral reasoning of those subjects. We might even discover that the self-abnegation frame plays some part in justice-related dictator-game scenarios, since it would seem to motivate suffering and self-denial. Ultimately, all four frames (excluding egoism) could be harnessed together as more effective appeals to inspire and crowd-in civic and altruistic behavior than any frame working on its own.

In addition to providing a clear measurement framework for philosophical moral framing, our findings can inform practical interventions for organizations and societies aiming to increase altruistic behavior. In Studies 1 and 2, deontic-virtue and utilitarian beliefs led to an increase in altruistic behavior, while egoism decreased it. This indicates that promoting a focus on others and the greater good, while at the same time shifting people’s state of mind away from self-interest is likely to increase such behavior. To gain insight on how to instill such a focus, we can turn to the organizational literature on self-other focus. For example, let us consider one considerately dominant variable that has consistently been shown to shift people’s mindsets towards themselves and their personal outcomes and away from those of others and the larger collective: Power.

People who feel a high sense of control over valued resources—over and above others—tend to focus on themselves to the detriment of those others. For example, Galinksy and colleagues [104] found that those primed to feel high power were more likely to draw images from their own perspective—even when they were aware a partner would see those images, were less likely to take into account information others had when making a decision, and were less accurate in determining other’s emotional expressions. Subsequent work has found that those who feel powerful—regardless of the resources they actually control—tend to ignore others’ input and perspectives when making ethical decisions [105], increase spending on products for themselves versus others [106], and give less to charity especially in low power distance cultures [107]. These results seem clear: People who feel powerful tend to focus on themselves, to the potential detriment of the greater good. Unfortunately for everyone, people in power have the greatest ability to affect the greater good.

Fortunately, a few clear interventions should help mitigate this egoism. First, since power is based on one’s dependence on the resources of another, interventions aimed to increase feelings of interdependence, or that others are necessary to complete common goals [108], should reduce feelings of dominance in the powerful and thus promote more virtue-ethical and utilitarian motivational states. A few of the most studied interventions involve emphasizing the capability of collectives to accomplish meaningful work and emphasizing one’s role within a larger collective, as opposed to their position atop it or more personal accomplishments [109]. Additionally, this literature identifies variables such as regulatory focus (promotion vs. prevention focus) that may increase focus on others [110–111] and the extensive literature on organizational citizenship behaviors has likewise identified several factors that may be manipulated to increase out-of-role helping for the greater good (e.g. [112]).

Still, it may not always be possible to turn those with a strong sense of egoism away from what they consider self-important. Study 3 provides us with one practical application of facing this prospect. Mainly, in Study 3, where we surveyed people participating in New York City’s Annual AIDS Walk and March of Dimes, egoism was positively related to charitable donation. Presumably, this was because the incentive structure of a charity environment publicly rewards those who raise the most for others. Thus, in the case of those with strong senses of egoism who refuse to consider themselves as part of a larger collective—as our previous suggestions entailed—it may be possible to either convince them that helping others is ultimately in their own self-interest or to change the context of a workplace or larger society to actually reward those who act for the greater good with encouragements such as praise, social status, and recognition. The latter would of course require a broad cultural shift and thus would likely not be immediately implementable. However, our findings strongly suggest that appealing to philosophical moral frames such as deontology-virtue and utilitarianism may often be more effective than instrumental appeals to power and social status in bringing about such changes.

## Conclusion

With the construction and initial validation of this Philosophical Moral-Framing Measure (PMFM) instrument, scholars now have a new tool to discern the actual motivations behind observed behavior, rather than simply relying on extrapolation and frames that are top-of-mind. We look forward to seeing continued work in this important space, and for future scholars to continue to explore the role of philosophical thinking in moral motivation. If, as our research would indicate, adult altruistic behavior may indeed be philosophical, further research is needed on how this thinking is developed and maintained. Such answers could hold the key to fostering more functional and sustainable organizations led by agents motivated by higher moral principles instead of mere instrumental aims of power and social status (e.g. [113]).

## Supporting information

S1 Data (SAV)

S2 Data (SAV)

S3 Data (SAV)

## References

[1] Cropanzano R., Goldman B., & Folger R. (2003). Deontic justice: The role of moral principles in workplace fairness. Journal of Organizational Behavior, 24, 1019–1024.

[2] Turillo C. J., Folger R., Lavelle J. J., Umphress E. E., & Gee J. O. (2002). Is virtue its own reward? Self-sacrificial decisions for the sake of fairness. Organizational Behavior and Human Decision Processes, 89, 839–865.

[3] Atiq E. H. (2014). Why motives matter: Reframing the crowding out effect of legal incentives. Yale Law Journal, 123(2), 862–1117.

[4] Bowles S. (2016). The Moral Economy: Why Good Incentives Are No Substitute for Good Citizens. New Haven, CT: Yale University Press.

[5] Sandel M. J. (2012). What Money Can't Buy: The Moral Limits of Markets. New York: Farrar, Straus and Giroux.

[6] Kahneman D., Knetsch J. L., & Thaler R. H. (1986). Fairness and the assumptions of economics. Journal of Business, 59, S285–S300.

[7] Friedland J., & Cole B. M. (2013). Expanding the motivations for altruism: A philosophical perspective. Journal of Organizational Behavior, 34(8), 1202–1206.

[8] Kohler S. (2011). Altruism and fairness in experimental decisions. Journal of Economic Behavior and Organization. 80(1), 101–109.

[9] Murphy R. O., Ackermann K. A., & Handgraaf M. (2011). Measuring social value orientation. Judgment and Decision making, 6(8), 771–781.

[10] Giacalone R. A., & Promislo M. D. (2013). Broken when entering: The stigmatization of goodness and business ethics education. Academy of Management Learning & Education, 12(1), 86–101.

[11] Kant E. (1785). Grundlegung zur Metaphysik der Sitten. Riga: Johan Friedrich Hartfnech.

[12] Mill J. S. (1863). Utilitarianism. London: Parker, Son, and Bourn, West Strand.

[13] Aristotle. (350 BCE). Aristotle's Nicomachean Ethics.

[14] Rothbard M. N. (2006). For a New Liberty: The Libertarian Manifesto. Auburn, AL: Ludwig von Mises Institute.

[15] Scanlon T. M. (2000). What We Owe to Each Other. Harvard.

[16] Batson C. D., Batson J. G., Griffitt C. A., Barrientos S., Brandt J. R., Sprengelmeyer P., et al (1989). Negative-state relief and the empathy-altruism hypothesis. Journal of Personality and Social Psychology, 56(6), 922–933.

[17] Granovetter M. (1985). Economic action and social structure: The problem of embeddedness. American Journal of Sociology, 91(3), 481–510.

[18] Cyert R. M., & March J. G. (1963). A behavioral theory of the firm. Englewood Cliffs, NJ: Prentice-Hall.

[19] Allison G. T. (1971). Essence of Decision: Explaining the Cuban Missile Crisis. Glenview, IL: Scott, Foresman and Co.

[20] Kahneman D., Slovic P., & Tversky A. (1982). Judgment under uncertainty: Heuristics and biases. Cambridge: Cambridge University Press. 10.1126/science.185.4157.1124 17835457

[21] Simon H. A. (1947). Administrative Behavior. New York: Macmillan Co.

[22] Piff P. K., Dietze P., Feinberg M., Stancato D. M., & Keltner D. (2015). Awe, the small self, and prosocial behavior. Journal of Personality and Social Psychology, 108(6), 883–899. 10.1037/pspi0000018 25984788

[23] Buchanan J. M., Tollison R. D., & Tullock G. (1980). Toward a theory of the rent-seeking society. College Station: Texas A & M University.

[24] Bowie N. (1991). Challenging the Egoistic Paradigm. Business Ethics Quarterly. 1(1), 1–21.

[25] Aldred J. (2010). The Skeptical Economist: Revealing the Ethics Behind Economics. Routledge.

[26] Ifcher J., & Zarghamee H. 2018 The rapid evolution of Homo Economicus: Brief exposure to neoclassical assumptions increases self-interested behavior. Journal of Behavioral and Experimental Economics, 75, 55–65.

[27] Pirson M. A., & Lawrence P. R. (2010). Humanism in business: Towards a paradigm shift? Journal of Business Ethics, 93(4), 553–565.

[28] Friedland J., & Cole B. M. (2017). From Homo-Economicus to Homo-Virtus: A system-theoretic model for raising moral self-awareness. Journal of Business Ethics, Advanced Online.

[29] Batson C. D. (1995). Prosocial motivation: Why do we help others? In Tesser A. (Ed.), Advanced Social Psychology (pp. 332–381). Boston, MA: McGraw-Hill.

[30] Piff P. K., Kraus M. W., Côté S., Cheng B., & Keltner D. (2010). Having less, giving more: The influence of social class on prosocial behavior. Journal of Personality and Social Psychology, 99, 771–784. 10.1037/a0020092 20649364

[31] Bloom P. (2016). Against Empathy: The Case for Rational Compassion. Ecco.

[32] Kinnick K. N., Krugman D. M., & Cameron G. T. (1996). Compassion fatigue: Communication and burnout towards social problems. Journalism and Mass Communications Quarterly, 73(3), 687–707.

[33] Dunlap R. E., Rosa E. A., Baxter R., & Mitchell R. (1993). Attitudes toward siting a high-level nuclear waste repository at Hanford, Washington In Dunlap R. E., Kraft M. E. & Rosa E. A. (Eds.), Public Reactions to Nuclear Waste. Durham, NC: Duke University Press.

[34] Gerber A. S., Green D. P., & Larimer C. W. (2008). Social pressure and voter turnout: Evidence from a large-scale field experiment. American Political Science Review, 102(1), 33–48.

[35] Kunreuther H., & Easterling D. (1996). The role of compensation in siting hazardous facilities. Journal of Policy Analysis and Management, 15(4), 601–622.

[36] Schwartz R. D., & Orleans S. (1967). On legal sanctions. University of Chicago Law Review, 34, 274–300.

[37] Aquino K., & Reed A. II (2002). The self-importance of moral identity. Journal of Personality and Social Psychology, 83(6), 1423–1440. 10.1037//0022-3514.83.6.1423 12500822

[38] Mazar N., Amir O., & Ariely D. (2008). The dishonesty of honest people: A theory of self-concept maintenance. Journal of Marketing Research, 45(6), 633–644.

[39] Lakoff G. (2002). Moral Politics: How Liberals and Conservatives Think. Chicago: Universituy of Chicago Press.

[40] Haidt J. (2012). The Righteous Mind: Why Good People Are Divided by Politics and Religion. New York: Random House.

[41] Dubljević V., Sattler S. & Racine E. (2018). Deciphering moral intuition: How agents, deeds, and consequences influence moral judgment. PLOS ONE, 13(10): e0204631 10.1371/journal.pone.0204631 30273370 PMC6166963

[42] Smith A. (1776). An inquiry into the nature and causes of the wealth of nations. London, [New York]: Printed for Strahan W. and Cadell T.; Kelley A. M.

[43] Nietzsche F. (1998). On the Geneology of Morality: A Polemic (Clark M. & Swensen A. J., Trans.). Indianapolis/Cambridge: Hackett Publishing Company, Inc.

[44] Parfit D. (2011). On What Matters. Oxford.

[45] Kant E. (2009). Groundwork of the Metaphysic of Morals (Paton H. J., Trans.). London: Harper Perennial Modern Classics.

[46] Rawls J. (2001). Justice as fairness: A restatement. Cambridge, MA: Harvard University Press.

[47] Kant E. (1974). Anthropology From a Pragmatic Point of View (Dowdell V. L., Trans.). The Hague, Netherlands: Martinus Nijhoff.

[48] O'Neil O. (1989). Constructions of Reason. Cambridge: Cambridge University Press.

[49] Bentham J. (1780). An Introduction to the Principles of Morals and Legislation. New York: Dover, 2007.

[50] Hume D. (1751). An Enquiry Concerning the Principle of Morals. London: A. Millar.

[51] Sidgwick H. (1884). The Methods of Ethics. London: Macmillan and Co.

[52] Singer P. (2011). Practical Ethics. Cambridge: Cambridge University Press.

[53] Brandt R. B. (1988). Fairness to indirect optimific theories in ethics. Ethics, 98(2), 341–360.

[54] Kahane G., Everett J. A. C., Earp B. D., Caviola L. Faber N. S., Crockett M. J., et al (2018). Beyond Sacrificial Harm: A Two-Dimensional Model of Utilitarian Psychology. Psychological Review, 125(2), 131–164. 10.1037/rev0000093 29265854 PMC5900580

[55] Aristotle. (2011). Aristotle's Nicomachean Ethics. Chicago, IL: University of Chicago Press.

[56] Hardie W. F. R. (1965). The final good in Aristotle's Ethics. Philosophy, 40(154), 277–295.

[57] Nowak M. A., Tarnita C. E., & Wilson E. O. (2010). The evolution of eusociality. Nature, 466(7310), 1057–1062. 10.1038/nature09205 20740005 PMC3279739

[58] Dawkins R. (1976). The Selfish Gene. Oxford: Oxford University Press.

[59] Stigler G. J., & Becker G. S. (1977). De gustibus non est disputandum The American Economic Review, 67(1), 76–90.

[60] Smith A. (1790). A Theory of Moral Sentiments. London: A. Millar.

[61] Conway D. W., & Groff P. S. (1998). Nietzsche: Critical Assessments (Vol. Vol. II 'The World as Will to Power—And Nothing Else?' Metaphysics and Epistemology). London: Routledge.

[62] Nietzsche F. (1962). Philosophy in the Tragic Age of the Greeks: Regnery Publishing, Inc.

[63] Boddy C. R. (2011). The corporate psychopaths theory of the global financial crisis. Journal of Business Ethics, 102(2), 255–259.

[64] Stout M. (2005). The sociopath next door: The ruthless versus the rest of us (1st ed). New York: Broadway Books.

[65] Colby A., Kohlberg L., Speicher B., Hewer A., Candee D., Gibbs J., et al (1987). The Measurement of Moral Judgement: Volume 2, Standard Issue Scoring Manual. Cambridge: Cambridge University Press.

[66] Vugta E., Asscher J., Jan Stams G., Hendriks J., Bijleveld C., & van der Laan P. (2011). Moral judgment of young sex offenders with and without intellectual disabilities. Research in Developmental Disabilities, 32(6), 2841–2846. 10.1016/j.ridd.2011.05.022 21689903

[67] Langdon P. E., Murphy G. H., Clare I. C. H., & Palmer E. J. (2010). The psychometric properties of the Socio-Moral Reflection Measure—Short Form and the Moral Theme Inventory for men with and without intellectual disabilities. Research in Developmental Disabilities, 31(6), 1204–1215. 10.1016/j.ridd.2010.07.025 20828987

[68] Rest J., Cooper D., Coder R., Masanz J., & Anderson D. (1974). Judging the important issues in moral dilemmas—an objective test of development. Developmental Psychology, 10, 491–501.

[69] Bebeau M. J. (2002). The defining issues test and the four component model: Contributions to professional education. Journal of Moral Education, 31(3), 271–295. 10.1080/0305724022000008115 15027443

[70] Kohlberg L. (1971). From is to ought: How to commit the naturalistic fallacy and get away with it in the study of moral development In Mischel T. (Ed.), Cognitive development and epistemology (pp. 151–235). New York: Academic Press.

[71] Punzo V. A. (1996). After Kohlberg: Virtue ethics and the recovery of the moral self. Philosophical Psychology, 9(1): 7–23.

[72] You D., & Bebeau M. (2013). The independence of James Rest’s components of morality: Evidence from a professional ethics curriculum study. Ethics and Education, 8(3), 202–216.

[73] Rand D., Briscell V., Everett J., Capraro V., Barcelo H. (2016). Social heuristics and social roles: Intuition favors altruism for women but not for men. Journal of Experimental Psychology, General, 145(4), 389–396. 26913619 10.1037/xge0000154

[74] Jackman S. (2001). Voting: Compulsory In Smelser N. J. & Baltes P. B. (Eds.), International encyclopedia of the social & behavioral sciences (pp. 16314–16318). Amsterdam: Elsevier.

[75] Frey B. S. (1997). A constitution for knaves crowds out civic virtues. The Economic Journal, 107(443), 1043–1053.

[76] Xing Y., Starik M. (2017). Taoist leadership and employee green behaviour: A cultural and philosophical microfoundation of sustainability, Journal of Organizational Behavior, 38, 9, (1302–1319).

[77] Singer P. (2015). The Most Good You Can Do: How Effective Altruism is Changing Ideas About Living Ethically. New Haven: Yale.

[78] Reidenbach R. E. & Robin D. P. (1990). Toward the development of a multidimensional scale for improving evaluations of business ethics. Journal of Business Ethics, 9(8):639–653.

[79] Hyman M. R. (1996). A critique and revision of the multidimensional ethics scale. Journal of Empirical Generalisations in Marketing Science, 1, 1–35.

[80] Furnham A., Humphries C., & Leung Zheng E. (2016). Can successful sales people become successful managers? Differences in motives and derailers across two jobs. Consulting Psychology Journal: Practice and Research, 68(3), 252.

[81] Shanahan K.J. & Hyman M.R. (2003). The development of a virtue ethics scale. Journal of Business Ethics, 42(2), 197–208.

[82] Hinkin, T. R. (1998). A brief tutorial on the development of measures for use in survey questionnaires. The Scholarly Commons, Cornel University School of Hotel Administration, Accessed Sept. 9, 2017: http://scholarship.sha.cornell.edu/articles/2521/.

[83] Ford J. K., MacCallum R. C., & Tait M. (1986). The application of exploratory factor analysis in applied psychology: A critical review and analysis. Personnel Psychology, 39(2), 291–314.

[84] Price J.L., & Mueller C.W. (1986) Handbook of organizational measurement. Marshfield, MA: Pitman.

[85] Nunnally J. C. (1978). Psychometric Theory. New York, NY: McGraw-Hill.

[86] Hayton J. C., Allen D. G., & Scarpello V. (2004). Factor retention decisions in exploratory factor analysis: A tutorial on parallel analysis. Organizational Research Methods, 7(2), 191–205.

[87] Tabachnick B. G. and Fidell L. S. (2014). Using Multivariate Statistics, 6th Edition Harlow: Pearson.

[88] Field A. (2013). Discovering Statistics using SPSS, 4th Edition London: SAGE.

[89] Paolacci G., Chandler J., & Ipeirotis P. G. (2010). Running experiments on Amazon Mechanical Turk. Judgment and Decision Making, 5(5), 411–419.

[90] Organ D. W., Podsakoff P. M., & MacKenzie S. B. (2005). Organizational citizenship behavior: Its nature, antecedents, and consequences. Sage Publications.

[91] Smith C. A., Organ D. W., & Near J. P. (1983). Organizational citizenship behavior: Its nature and antecedents. Journal of Applied Psychology, 68(4), 653–663.

[92] Dierksmeier C. (2013). Kant on virtue. Journal of Business Ethics, 113(4), 597–609.

[93] Piaget J. (1975). L’équilibration des structures cognitives: Problème central du développement. Paris: PUF.

[94] Yeager L. B. (2002). Ethics as Social Science: The Moral Philosophy of Social Cooperation. Northampton, MA: Edward Elgar 105–107.

[95] Jones D. N., & Paulhus D. L. (2014). Introducing the Short Dark Triad (SD3): A brief measure of dark personalities. Assessment, 21(1), 28–41. 10.1177/1073191113514105 24322012

[96] Myrseth K. O. R., Fishbach A., & Trope Y. (2009). Counteractive self-control: When making temptation available makes temptation less tempting. Psychological Science, 20(2), 159–163. 10.1111/j.1467-9280.2009.02268.x 19170939

[97] Tronto J. C. (1987). Beyond gender difference to a theory of care. Signs: Journal of Women in Culture and Society, 12(4), 644–663.

[98] Crowne D. P., & Marlowe D. (1960). A new scale of social desirability independent of psychopathology. Journal of Consulting Psychology, 24(4), 349–354. 13813058 10.1037/h0047358

[99] Joinson A. (1999). Social desirability, anonymity, and internet-based questionnaires. Behavioral Research Methods, Instruments & Computers, 31(3), 433–438. 10.3758/bf03200723 10502866

[100] Piff P. K., Stancato D. M., Côté S., Mendoza-Denton R., & Keltner D. (2012). Higher social class predicts increased unethical behavior. Proceedings of the National Academy of Sciences, 109(11), 4086–4091. 10.1073/pnas.1118373109 PMC3306667 22371585

[101] Kraus M. W., Côté S., & Keltner D. (2010). Social class, contextualism, and empathic accuracy. Psychological Science, 21(11), 1716–1723. 10.1177/0956797610387613 20974714

[102] Awad E., Dsouza S., Kim R., Schulz J., Henrich J., Shariff A., et al (2018). The Moral Machine Experiment. Nature, 563, 59–64. 10.1038/s41586-018-0637-6 30356211

[103] Piazza J., & Sousa P. (2014). Religiosity, political orientation, and consequentialist moral thinking. Social Psychological and Personality Science, 5(3), 334–342.

[104] Galinsky A. D., Magee J. C., Inesi M. E., & Gruenfeld D. H. (2006). Power and perspectives not taken. Psychological Science, 17, 1068–1074. 10.1111/j.1467-9280.2006.01824.x 17201789

[105] Pitesa M., & Thau S. (2013). Compliant sinners, obstinate saints: How power and self-focus determine the effectiveness of social influences in ethical decision making. Academy of Management Journal, 56(3), 635–658.

[106] Rucker Derek D., Dubois David, and Galinsky Adam D. (2010). Generous paupers and stingy princes: Power drives consumer spending on self versus others. Journal of Consumer Research 37(6), 1015–1029.

[107] Han D., Lalwani A. K., & Duhachek A. (2017). Power Distance Belief, Power, and Charitable Giving. Journal of Consumer Research, 44(1), 182–195.

[108] Gully S. M., Incalcaterra K. A., Joshi A., & Beaubien J. M. (2002). A meta-analysis of team-efficacy, potency, and performance: interdependence and level of analysis as moderators of observed relationships. Journal of Applied Psychology, 87(5), 819–832. 10.1037/0021-9010.87.5.819 12395807

[109] Emich K. J. (2012). How expectancy motivation influences information exchange in small groups. Small Group Research, 43(3), 275–294.

[110] Lin S. H. J., & Johnson R. E. (2018). Opposing Affective and Cognitive Effects of Prevention Focus on Counterproductive Work Behavior. Journal of Business and Psychology, 33, 283–296.

[111] Polman E. (2012). Effects of self–other decision making on regulatory focus and choice overload. Journal of Personality and Social Psychology, 102(5), 980–993. 10.1037/a0026966 22429272

[112] Dalal R. S. (2005). A meta-analysis of the relationship between organizational citizenship behavior and counterproductive work behavior. Journal of Applied Psychology, 90(6), 1241–1255. 10.1037/0021-9010.90.6.1241 16316277

[113] Djurdjevic E., Stoverink A. C., Klotz A. C., Koopman J., da Motta Veiga S. P., Yam K. C., et al (2017). Workplace status: The development and validation of a scale. Journal of Applied Psychology, Advanced online. 10.1037/apl0000202 28333498

